# Effect of the Addition of Varying Concentrations of Silver Nanoparticles on the Fluoride Uptake and Recharge of Glass Ionomer Cement

**DOI:** 10.3390/nano12121971

**Published:** 2022-06-08

**Authors:** Turki D. Alshehri, Sunil Babu Kotha, Faisal Mohammed Abed, Mohammed J. Barry, Abdulrahman AlAsmari, Sreekanth Kumar Mallineni

**Affiliations:** 1 Ministry of Health, Abha Maternity and Children Hospital, Abha 62562, Saudi Arabia; turkialshehri@windowslive.com; 2 Preventive Dentistry Department, Pediatric Dentistry Division, College of Dentistry, Riyadh Elm University (REU), Riyadh 13244, Saudi Arabia; Arabia.faisalabed2011@gmail.com (F.M.A.); dr.m.j.barry@gmail.com (M.J.B.); aldrwas91@gmail.com (A.A.); 3 Department of Pediatric and Preventive Dentistry, Sharad Pawar Dental College and Hospital, Datta Meghe Institute of Medical Sciences (Deemed to be University), Wardha 442004, India; 4 Ministry of Health Specialized Dental Center, King Fahd General Hospital, Madinah 42315, Saudi Arabia; 5 Ministry of Health, Specialized Dental Center, Ohod Hospital, Madinah 42315, Saudi Arabia; 6 Ministry of Health, Al Qunfudah Dental Center, Al Qunfudah 28821, Saudi Arabia; 7 Department of Preventive Science, College of Dentistry, Majmaah University, Almajmaah 11952, Saudi Arabia; 8 Center for Transdisciplinary Research (CFTR), Saveetha Institute of Medical and Technical Sciences, Saveetha Dental College, Saveetha University, Chennai 600077, India

**Keywords:** silver nanoparticles, fluoride, glass ionomer cement

## Abstract

This study aimed to compare the amount of fluoride uptake and the recharge and release characteristics of conventional glass ionomer cement (GIC) without any additives in comparison to conventional glass ionomer cement supplemented with silver nanoparticles (AgNPs) at two concentrations: 0.1% and 0.2% (*w*/*w*). A total of 60 specimens were used in this in vitro study. The sample was divided into six groups—including three groups without fluoride charge: Group 1 (conventional GIC), Group 2 (GIC with 0.1% silver nanoparticles), and Group 3 (GIC with 0.2% silver nanoparticles; and three groups with fluoride charge: Group 4 (conventional GIC with fluoride); Group 5 (GIC with 0.1% silver nanoparticles with fluoride); Group 6 (GIC with 0.2% silver nanoparticles with fluoride), where Group 1 is considered the control group and the other five groups are used as the test groups. The amount of fluoride released was measured on days 1, 2, 7, 15, and 30. The comparisons were made between the groups with and without fluoride and among all the groups. A significant difference in the amount of fluoride released was observed between the groups, with the highest amount occurring in Group 1, followed by Group 2; the lowest amount of fluoride released was observed in Group 3 (*p* < 0.05). The groups with fluoride recharge (Groups 4, 5, and 6) exhibited a higher amount of fluoride release than the groups with no recharge (Groups 1, 2, and 3); however, Group 1 has more fluoride release compared to all other groups on days 1, 2, 7, 15, and 30 (*p* < 0.05). The amount of released fluoride decreased from day 1 to day 30 in all of the groups in the study. Despite the antimicrobial and anticariogenic benefits of adding silver nanoparticles to GIC, it seems that fluoride release characteristics are significantly affected by the addition of this material. This may force the clinician to a compromise between the antimicrobial benefit of silver nanoparticles and the remineralizing advantage of fluoride.

## 1. Introduction

Having a caries-inhibiting property is considered to be very desirable in restorative materials [1]. Fluoride can play a vital role in the prevention of caries; thus, many efforts have been made to incorporate fluoride into distinct preventive materials [2,3,4]. Glass ionomer cement (GIC) has been developed as a more biocompatible alternative to silicate cement [5]. Fluoride has been used as a flux for reducing the glass fusion temperature throughout the manufacturing process as it imparts the natural property of fluoride release to the cement [6,7]. Studies that investigated the pattern of fluoride release in a restorative material in the 1990s reported that if the GICs were exposed to a fluoride source, then they would imbibe fluoride ions and therefore act as a “fluoride reservoir” [7,8,9]. The most well-known fluoride source that is normally used every day is fluoridated dentifrices; for this reason, fluoridated toothpaste has been used to measure fluoride recharge and uptake [10,11]. The concentration of fluoride in toothpaste can range from a low value, such as 500 ppm for a toothpaste for children, to a high value, such as 5000 ppm, in high-fluoride toothpaste [12]. Silver has an elementary and ionized form in silver nanoparticles or zeolites [13]. Silver alloy powder can be added to restorative glass ionomer cement to make reinforced GIC which is considered harder and stronger. In cermet cement, the process of sintering silver powder to glass at a high temperature can increase durability and enhance abrasion resistance [14,15].

Incorporating silver nanoparticles into GIC powder could inhibit biofilm formations whilst having almost no significant effects on any mechanical and physical properties. It has been reported that silver nanoparticles did not firmly bond with the matrix and thus, did not significantly improve its mechanical properties [16]. This could be a result of its nano-sized particles that can be dispersed around and among polymer chains [16]. Typically, in addition to the fact that GIC contains fluoride that is released into the oral environment when saliva is present, it could also be recharged by toothpaste gels or mouth rinses that contain fluoride [17]. However, there is little information in the literature regarding the fluoride uptake and recharge abilities of glass ionomer types of cement that are reinforced by silver nanoparticles [2,18]. The ability of glass ionomer cement to recharge is likely a result of their capability to re-release ions from a solution which might permit their application as “rechargeable reservoirs” for ion distributions that include fluoride. Therefore, the majority of studies that have investigated fluoride ion uptake were concerned with solution ion concentrations and carried out analyses pre-and post-GIC immersion [19,20,21].

The study that was carried out by Arbabzadeh-Zavareh et al. [22] compared the recharge patterns of six different types of glass ionomer cement after fluoride exposure. Mouthwash and toothpaste products that contain fluoride were used. The authors reported that applying fluoride materials using a timetabled schedule could achieve high fluoride releases [18,22]. The current study aimed to evaluate the effects of different silver nanoparticle concentrations on the fluoride uptake and recharge properties of conventional glass ionomer cement. It compares the amount of fluoride uptake and the recharge and release characteristics of conventional glass ionomer cement (GIC) without any additives to conventional glass ionomer cement that is supplemented with silver nanoparticles (AgNPs) at two concentrations: 0.1% and 0.2% (*w*/*w*).

## 2. Materials and Methods

### 2.1. Ethical Approval

This work was registered with the research center of Riyadh Elm University (FPGRP/43835002/342) and ethical approval was obtained from the Institutional Review Board (IRB) of the institution.

### 2.2. Study Design

This study followed an experimental design using a fluoride electrode to measure the three cements’ fluoride uptake and recharge characteristics.

### 2.3. Material and Devices

A conventional GIC (GC Fuji II, GC Corporation, Tokyo, Japan) was used in this study. Silver nano powder with a <100 nm particle size (Sigma-Aldrich Co., St. Louis, MO, USA) was purchased and added to the powder of the GIC. Specimens were prepared with 2 different concentrations of silver (0.1% and 0.2% (*w*/*w*)) using an electronic weighing scale. The AgNP powder was carefully weighed using a weighing machine with an accuracy of ±0.0001 g (A&D, GR + 360, Tokyo, Japan). The GIC specimens were divided into three groups for each test: GIC without AgNPs; GIC with 0.1% AgNPs; GIC with 0.2% AgNPs. The fillings were mixed at a P/L ratio of 2.6/1 g and were prepared following the manufacturer’s instructions.

### 2.4. Preparation of the Sample

A total of 60 specimens (10 in each group) were used in this study. The 10 specimens in each group were further subdivided into 6 subgroups of 10 each. No fluoride treatment was applied to the groups (Groups 1, 2, and 3) and fluoride treatment was given to 3 other groups (Groups 4, 5, and 6). The molds were prepared with 6 mm in diameter and 3 mm in height. Excess materials were removed and dental floss (for suspension in the solution) was imbedded into each specimen. For the second subgroup, a 1450 ppm dentifrice was applied for 2 min, twice daily, with a soft toothbrush used for the specific group. The specimens were then suspended in airtight plastic bottles containing exactly 20 mL of double-deionized water.

**Thus, the groups were as follows:**

Group 1 (10 specimens): control group with no recharge;
Group 2 (10 specimens): 0.1% silver nanoparticles with no recharge;
Group 3 (10 specimens): 0.2% silver nanoparticles with no recharge;
Group 4 (10 specimens): control group with fluoride recharge;
Group 5 (10 specimens): 0.1% silver nanoparticles with fluoride recharge;
Group 6 (10 specimens): 0.2% silver nanoparticles with fluoride recharge.

### 2.5. Measurement of Fluoride Uptake and Recharge

The fluoride content and its distribution in all the samples were analyzed using a fluoride ion selective electrode (HI4110 Fluoride ISE, Solid-state Combination, Hanna Instruments Co., Carrollton, TXUSA) connected to an ion selective electrode meter/digital ion analyzer. The total ionic strength adjustment buffer (TISAB) was added to all water specimens to maintain the pH between 5.0 and 5.5. The fluoride electrode was calibrated using a sodium fluoride stock solution with a concentration of 100 ppm fluoride. This solution was then diluted, in stages, with double-distilled water to produce standard solutions of 20 ppm, 10 ppm, 5 ppm, and 2.5 ppm fluoride. Fluoride measurements were recorded at intervals of days 1, 2, 7, 15, and 30. The electrode was recalibrated at every interval.

### 2.6. Statistical Analysis

All analyses were performed using Statistical Package for Social Sciences (SPSS) software Version 21.0 (Armonk, NY, USA, IBM Corp). The descriptive statistics mean and SD for continuous data and the median (interquartile range (IQR)) for non-normally distributed interval data and ordinal data were presented. Based on the normality of data, the parametric t-test and non-parametric Mann–Whitney U test were applied to the data to find differences between the two groups. The non-parametric Kruskal–Wallis test was used to compare three groups. The Shapiro–Wilk and histogram with summary values were used to test the hypothesis of normal distribution.

## 3. Results

**Figure 1:**
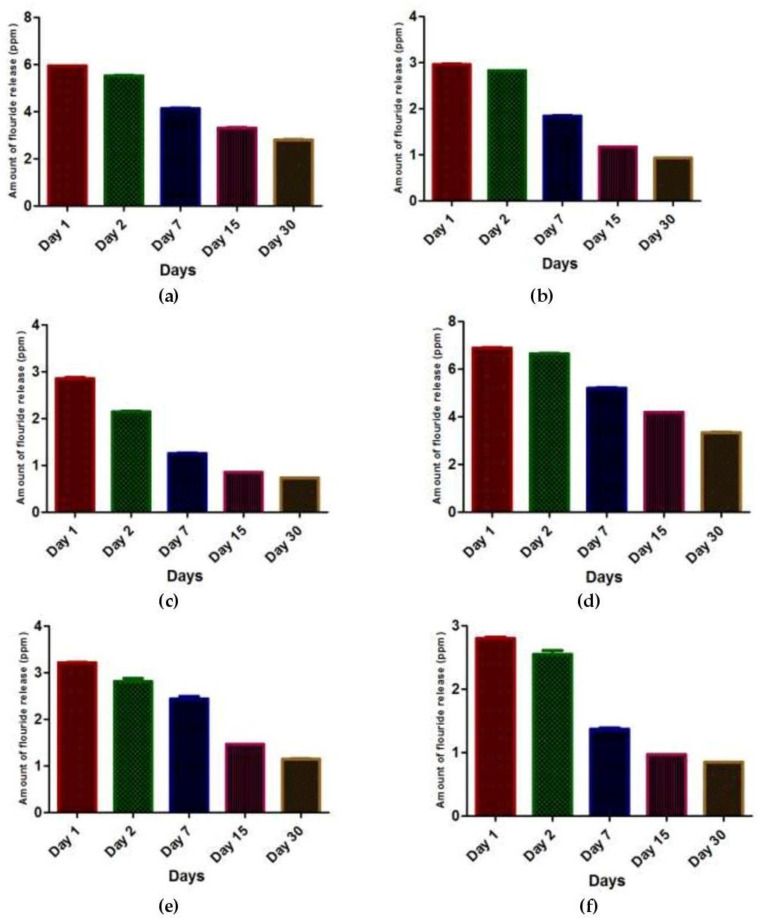
Mean values of fluoride release in (**a**) Group 1 (control group with no recharge); (**b**) Group 2 (0.1% silver nanoparticles with no recharge); (**c**) Group 3 (0.2% silver nanoparticles with no recharge); (**d**) Group 4 (control group with fluoride recharge); (**e**) Group 5 (0.1% silver nanoparticles with fluoride recharge); and (**f**) Group 6 (0.2% silver nanoparticles with fluoride recharge) on days 1, 2, 7, 15, and 30.

**Figure 2:**
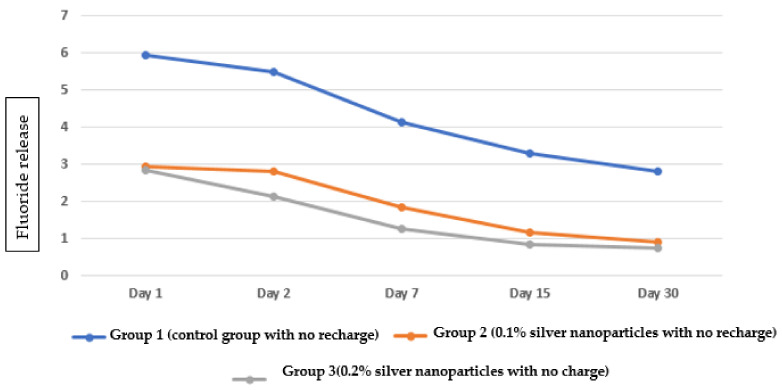
Pattern of fluoride release from the three groups at different time intervals without fluoride recharge.

The fluoride release in Group 1 (control group with no recharge) gradually decreased over time with the highest amount on day 1 and the lowest on day 30 (Figure 1a). Fluoride release in Group 2 (0.1% silver nanoparticles with no recharge) gradually decreased over time, with the highest amount on day 1 and the lowest on day 30 (Figure 1b). Fluoride release in Group 3 (0.2% silver nanoparticles with no recharge) gradually decreased over time, with the highest amount on day 1 and the lowest on day 30 (Figure 1c). Regarding the pattern of fluoride release from the three groups without fluoride recharge, the highest fluoride release was observed in Group 1, followed by Group 2, and the lowest amount of fluoride release was observed in Group 3 (Figure 2).

Fluoride release in Group 4 (control group with fluoride recharge) gradually decreased over time, with the highest amount on day 1 and the lowest on day 30 (Figure 1d).

**Figure 3:**
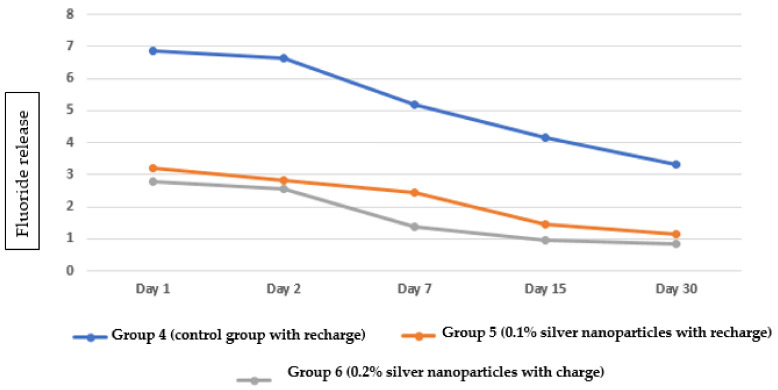
Pattern of fluoride release from the three groups at different time intervals with fluoride recharge.

Fluoride release in Group 5 (0.1% silver nanoparticles with fluoride recharge) gradually decreased over time with the highest amount on day 1 and the lowest on day 30 (Figure 1e). Fluoride release in Group 6 (0.2% silver nanoparticles with fluoride recharge) gradually decreased over time, with the highest amount on day 1 and the lowest on day 30 (Figure 1f). Regarding the pattern of fluoride release from the three groups with fluoride recharge, the highest fluoride release was observed in Group 4, followed by Group 5, and the lowest amount of fluoride release was observed in Group 6 (Figure 3).

None of the comparisons between the baseline and baseline groups with fluoride release during the studied periods showed statistical significance. (*p* > 0.05). The comparison among the no charge (Groups1,2, and 3) and with charge (Groups (4,5, and 6) were shown in Table 1. In all the baseline (no charge) groups on 1st, 2nd, 7th, 15th, and 30th days fluoride release was comparatives more than charge groups (*p* > 0.05). The comparison between the three groups without fluoride recharge (Group 1 (control group with no recharge); Group 2 (0.1% silver nanoparticles with no recharge); Group 3 (0.2% silver nanoparticles with no recharge)) showed statistically significant differences between groups (*p* < 0.05). The highest amount of fluoride release was observed in Group 1, followed by Group 2.0, and the lowest value was observed in Group 3 (Table 2). The comparison between the three groups with fluoride recharge (Group 4 (control group with no recharge); Group 5 (0.1% silver nanoparticles with recharge); Group 6 (0.2% silver nanoparticles with recharge)) showed statistically significant differences between the groups (*p* < 0.05). The highest amount of fluoride release was observed in Group 4, followed by Group 5, and the lowest value was observed in Group 6 (Table 2). The comparison between fluoride recharge and no recharge in each group (Group 1 (control group); Group 2 (); Group 3 found a statistically significant difference between Group 1 and Group 4 (*p* > 0.05), while the other groups showed no statistically significant differences in each pair (*p* > 0.05) (Table 2).

## 4. Discussion

The role of glass ionomer as a fluoride reservoir and its inherent ability to recharge fluoride has long been recognized [23]. This means that glass ionomer has a unique advantage over other dental materials. The present study was conducted to compare the amount of fluoride uptake and the recharge and release characteristics of conventional glass ionomer cement (GIC) without any additives compared to conventional glass ionomer cement that was supplemented with silver nanoparticles (AgNPs) at two concentrations: 0.1% and 0.2% (*w*/*w*). The fluoride ion selective electrode method was a reliable and accurate method to measure the amount of fluoride ions that were leached from the glass ionomer. This method has been used in numerous in vitro studies to accurately evaluate and compare fluoride release in different fluoride-releasing restorative materials [24,25,26,27].

The fluoride release characteristics of glass ionomer have been investigated in several studies [24,28,29,30]. These studies clearly showed that the fluoride release characteristics of glass ionomers were inversely proportional to the number of fillers added regardless of their type [24,31]. These findings are in agreement with the results observed in the present study and the authors found that the higher the concentration of silver nanoparticles was, the lower the amount of fluoride released was. A possible explanation for this is that fillers (regardless of their type) are not soluble and possess very good mechanical properties in comparison to the glass ionomer matrix, which is the medium that is responsible for fluoride release. Thus, increasing the amount of fillers in glass ionomer will eventually decrease the amount of the matrix that releases the fluoride and decrease the fluoride release. However, the relationship between the amount of fluoride inside each cement and the ability of this cement to release the fluoride inside is still not well understood. It was found that three main mechanisms can explain fluoride release from glass ionomers, including the diffusion of ions through pores by superficial rinsing; microfractures on the surface, which enable fluoride ion leaching; and, finally, the mass diffusion concept [31,32].

This provides silver nanoparticles with an important anticariogenic effect at low toxicity [33,34,35]. Furthermore, previous studies have found that the incorporation of silver nanoparticles did not affect the cytotoxicity of human cells. The antibacterial activity of silver nanoparticles that have been incorporated into glass ionomer cement may last for very long periods—up to 4 months [36]. Therefore, it is important to evaluate their effect on fluoride recharge and release because the fluoride releasing ability of glass ionomers is also an important property that the authors do not want to compromise, due to its importance in remineralizing carious cavities. The findings from the present study show that fluoride release was significantly affected by the incorporation of silver nanoparticles. Based on these observations, the authors opined that a higher concentration of silver nanoparticles means a lower fluoride release is added to the glass ionomer cement. This may put the clinician in a situation in which they must make a compromise between the strong antimicrobial effect of silver nanoparticles incorporated with glass ionomers and the remineralizing effect of conventional glass ionomers due to their significant fluoride-releasing ability. Furthermore, it must be understood that a sustained amount of fluoride release is needed [37].

Prior studies reported that the release of fluoride from glass ionomer cement follows an exponential pattern rather than a linear pattern, with the maximum amount of release occurring in the first and second days of its restoration in the oral cavity, which significantly decreases until it reaches the lowest amount on day 30 [24,38,39,40]. A similar observation was evident in the present study and the difference was considerably not high. These observations can be explained by the emptying of the fluoride from the glass ionomer matrix over time as, in the beginning, the glass ionomer matrix is fully recharged by fluoride which leaks out of the matrix until it is depleted. An exponential pattern of fluoride ion release is not ideal because, in order to maximize the benefit of the fluoride-releasing property, the authors must obtain a sustained level of fluoride release; this will not only help in terms of caries inhibition, but will also help to maintain the mechanical and physical properties of the glass ionomer for longer periods. Additionally, the authors found that fluoride recharging had a significant effect on the amount of fluoride released in the conventional glass ionomer groups, favoring recharge over no recharge. On the other hand, at concentrations of 0.1% and 0.2%, silver nanoparticles showed small differences between the recharged groups and at concentrations of 0.1% and 0.2%, silver nanoparticles showed small differences between the recharged and non-recharged groups, although the effect was statistically non-significant. It was also noted that the higher the concentration of silver nanoparticles was, the lower the difference was between the recharged and non-charged groups was. The rationale behind these results is clear: the authors cannot expect a material such as glass ionomer to release or recharge a significant amount of fluoride when it is loaded with silver nanoparticles. These cements will likely lack a sufficient amount of fluoride-containing matrix [41] and they will also lack space for fluoride recharge.

The findings observed in the present study are different from those that were identified in an analogous study conducted by Bamoussa et al. [24]. In the study by Bamoussa et al., a comparison was made between the characteristics and fluoride recharge and release of a zinc-reinforced glass ionomer cement to that of two traditional glass ionomer cements. Bamoussa et al. reported that the zinc-reinforced glass ionomer had a higher fluoride recharge and release that significantly differed from the two traditional glass ionomer cements [24]. To clarify this, it should be understood that silver nanoparticles have different characteristics to zinc. The effect of zinc on solubility is an enhancing effect rather than a restraining effect. This is due to the ease of ionization and the solubility of zinc particles inside the glass ionomer [42]. In contrast, silver nanoparticles are somewhat more resistant to solubility, thus restraining the ability of glass ionomers to release and recharge fluoride ions [43]. However, the clinical relevance of the laboratory testing protocols may be in question, as it was found that the mechanical loading of the glass ionomer could affect its ability to release and recharge fluoride by inducing microcracks in the matrix, thus enhancing the solubility of this material [44,45].

Other factors may affect the amount of release and recharge of fluoride ions (such as the temperature and degree of acidity of the solution), as it was found that, the higher the temperature and acidity were, the higher the fluoride ion release and the lower the ability of the material to recharge. Of course, in the present study, both the temperature and the pH were standardized in all of the samples. The pH in the present study was set between 5.0 and 5.5, which is considered acidic and may enhance fluoride ion release. This pH may significantly differ from clinical situations, as the pH in the oral cavity is above 5.5 most of the time (which is the critical pH for the demineralization of dental structures). Nevertheless, setting the pH to this level was necessary in the present study to obtain the extreme outcome of the material. Moreover, similar situations were also used in other studies measuring fluoride release, as this will eventually simplify future comparisons [24,37,46]. The results of the present study confirm that glass ionomers can release and recharge fluoride, regardless of whether or not silver nanoparticles were incorporated. Nevertheless, the amount of release and recharge of glass ionomers is significantly affected by the addition of silver nanoparticles, which can impact the remineralizing effect of this material [47,48].

### Limitations and Recommendations

The present study has some shortcomings. For instance, it is an in vitro study and it is well known that oral cavity conditions are not the same as laboratory conditions. Mechanical loading was not assessed in the present study, which is also a limitation. Additionally, the fluoride release measurements of glass ionomers into different storage media with different temperatures and pH values were not obtained. In addition, fluoride release characteristics under cyclic loading were not evaluated.

## 5. Conclusions

Despite the antimicrobial and anticaries benefits of adding silver nanoparticles to GIC, the results showed that the fluoride release characteristics were significantly negatively affected by the addition of this material. This may force the clinician to compromise between the antimicrobial benefit of silver nanoparticles and the remineralizing advantage of fluoride. The study demonstrated that there is a significant negative effect on the fluoride release characteristics of GIC with the addition of AgNPs, which means that the null hypothesis is not accepted. According to the findings of this study, it appears that the addition of silver nanoparticles with a concentration of 0.1% showed a reasonable amount of fluoride recharge and release in glass ionomers without the significant eradication of this advantage, thus combining the remineralizing advantage of fluoride and the antimicrobial effect of silver nanoparticles. Nevertheless, further studies are recommended to assess the antibacterial effect of these concentrations.

## Figures and Tables

**Table 1:** Comparison baseline and baseline groups with fluoride release.

Time Period	Group 1 Median (IQR)	Group 4 Median (IQR)	*p* Value	Group 2 Median (IQR)	Group 5 Median (IQR)	*p* Value	Group 3 Median (IQR)	Group 6 Median (IQR)	*p* Value
**Day 1**	5.21 (3.75,6.71)	4.12 (3.09,5.76)	0.251	2.31 (1.28,3.10)	1.87 (1.05,2.87)	0.465	1.34 (0.88,2.79)	1.27 (0.79,2.51)	0.754
**Day 2**	5.18 (3.77,6.70)	4.09 (3.09,5.70)	0.251	2.27 (1.26,3.12)	1.84 (1.04,2.87)	0.465	1.15 (0.85,2.38)	1.07 (0.76,1.93)	0.564
**Day 7**	5.14 (3.71,6.70)	4.10 (3.09,5.75)	0.347	2.29 (1.33,3.06)	1.89 (1.05,2.90)	0.602	1.12 (0.85,2.38)	1.04 (0.76,1.93)	0.564
**Day 15**	5.16 (3.74,6.69)	4.12 (3.05,5.69)	0.347	2.30 (1.25,3.07)	1.85 (1.03,2.87)	0.530	1.16 (0.85,2.37)	1.02 (0.76,1.89)	0.564
**Day 30**	5.20 (3.77,6.72)	4.08 (3.02,5.75)	0.251	2.25 (1.31,3.07)	1.79 (2.87,1.06)	0.530	1.12 (0.85,1.97)	1.05 (0.77,1.88)	0.564
**Total**	5.17 (3.75,6.71)	4.10 (3.07,5.73)	0.251	2.28 (1.29,3.09)	1.85 (1.05,2.87)	0.465	1.14 (0.85,2.30)	1.05 (0.76,1.91)	0.564

**Table 2:** Comparison of all study groups using Kruskal–Wallis test.

Time Period	Group 1 Median (IQR)	Group 2 Median (IQR)	Group 3 Median (IQR)	Group 4 Median (IQR)	Group 5 Median (IQR)	Group 6 Median (IQR)	*p* Value
**Day 1**	5.21 (3.75,6.71)	2.31 (1.28,3.10)	1.34 (0.88,2.79)	4.12 (3.09,5.76)	1.87 (1.05,2.87)	1.27 (0.79,2.51)	0.002 *
**Day 2**	5.18 (3.77,6.70)	2.27 (1.26,3.12)	1.15 (0.85,2.38)	4.09 (3.09,5.70)	1.84 (1.04,2.87)	1.07 (0.77,1.93)	0.001 *
**Day 7**	5.14 (3.71,6.70)	2.29 (1.33,3.06)	1.12 (0.85,2.38)	4.10 (3.09,5.75)	1.89 (1.05,2.90)	1.04 (0.76,1.93)	0.001 *
**Day 15**	5.16 (3.74,6.69)	2.30 (1.25,3.07)	1.16 (0.85,2.37)	4.12 (3.05,5.69)	1.85 (1.03,2.87)	1.02 (0.76,1.89)	0.002 *
**Day 30**	5.20 (3.77,6.72)	2.25 (1.31,3.07)	1.12 (0.86,1.97)	4.08 (3.02,5.75)	1.79 (1.06,2.87)	1.05 (0.77,1.88)	0.001 *
**Total**	5.17 (3.74, 6.71)	2.28 (1.28,3.08)	1.14 (0.85,2.30)	4.10 (3.07,5.73)	1.84 (1.05,2.87)	1.05 (0.76,1.91)	0.001 *

* Significant.

## References

[1] Rothwell M., Anstice H., Pearson G. (1998). The uptake and release of fluoride by ion-leaching cements after exposure to toothpaste. J. Dent..

[2] Mickenautsch S., Mount G., Yengopal V. (2011). Therapeutic effect of glass-ionomers: An overview of evidence. Aust. Dent. J..

[3] Trairatvorakul C., Itsaraviriyakul S., Wiboonchan W. (2010). Effect of Glass-ionomer Cement on the Progression of Proximal Caries. J. Dent. Res..

[4] Ngo H. (2010). Glass-Ionomer Cements as Restorative and Preventive Materials. Dent. Clin. N. Am..

[5] Arita K., Yamamoto A., Shinonaga Y., Harada K., Abe Y., Nakagawa K., Sugiyama S. (2011). Hydroxyapatite particle char-acteristics influence the enhancement of the mechanical and chemical properties of conventional restorative glass ionomer cement. Dent. Mater. J..

[6] Attar N., Turgut M.D. (2003). Fluoride release and uptake capacities of fluoride-releasing restorative materials. Oper. Dent..

[7] Young A., Von Der Fehr F.R., Sønju T., Nordbø H. (1996). Fluoride release and uptake in vitro from a composite resin and two orthodontic adhesives. Acta Odontol. Scand..

[8] AlJefri G.H., Kotha S.B., Murad M.H., Aljudaibi R.M., Almotawah F.N., Mallineni S.K. (2022). Penetration and Adaptation of the Highly Viscous Zinc-Reinforced Glass Ionomer Cement on Contaminated Fissures: An In Vitro Study with SEM Analysis. Int. J. Environ. Res. Public Health..

[9] Preston A.J., Higham S.M., Agalamanyi E.A., Mair L.H. (1999). Fluoride recharge of aesthetic dental materials. J. Oral Rehabil..

[10] Krämer N., Schmidt M., Lücker S., Domann E., Frankenberger R. (2018). Glass ionomer cement inhibits secondary caries in an in vitro biofilm model. Clin. Oral. Investig..

[11] Donly K.J., Nelson J.J. (1997). Fluoride release of restorative materials exposed to a fluoridated dentifrice. ASDC J. Dent. Child.

[12] Gatti A., Camargo L.B., Imparato J.C.P., Mendes F.M., Raggio D.P. (2011). Combination effect of fluoride dentifrices and varnish on deciduous enamel demineralization. Braz. Oral Res..

[13] Monteiro D.R., Gorup L.F., Takamiya A.S., de Camargo E.R., Filho A.C.R., Barbosa D.B. (2011). Silver Distribution and Release from an Antimicrobial Denture Base Resin Containing Silver Colloidal Nanoparticles. J. Prosthodont..

[14] Simmons J.J. (1983). The miracle mixture. Glass ionomer and alloy powder. Tex. Dent. J..

[15] Mclean J.W. (1990). Cermet cements. J. Am. Dent. Assoc..

[16] El-Wassefy N.A., El-Mahdy R.H., El-Kholany N.R. (2017). The impact of silver nanoparticles integration on biofilm formation and mechanical properties of glass ionomer cement. J. Esthet. Restor. Dent..

[17] Nicholson J.W., Czarnecka B. (2012). Maturation affects fluoride uptake by glass-ionomer dental cements. Dent. Mater..

[18] Sidhu S.K., Nicholson J.W. (2016). A Review of Glass-Ionomer Cements for Clinical Dentistry. J. Funct. Biomater..

[19] Wiegand A., Buchalla W., Attin T. (2007). Review on fluoride-releasing restorative materials-fluoride release and uptake char-acteristics, antibacterial activity and influence on caries formation. Dent. Mater..

[20] Gandolfi M.G., Chersoni S., Acquaviva G., Piana G., Prati C., Mongiorgi R. (2006). Fluoride release and absorption at different pH from glass-ionomer cements. Dent. Mater..

[21] Hadley P.C., Billington R.W., Pearson G.J. (1999). Effect of monovalent ions in glass ionomer on their uptake and re-release. Biomaterials.

[22] Arbabzadeh-Zavareh F., Meyers I., Mortazavi S., Gibbs T., Bouzari M., Walsh L. (2012). Recharge pattern of contemporary glass ionomer restoratives. Dent. Res. J..

[23] Thangavelu L., Adil A.H., Arshad S., Devaraj E., Mallineni S.K., Sajja R., Chakradhar A., Karobari M.I. (2021). Antimicrobial Properties of Silver Nitrate Nanoparticle and Its Application in Endodontics and Dentistry: A Review of Literature. J. Nanomater..

[24] Bamoussa A.A., Assery M.K., Pani S.C. (2015). Fluoride release and recharge abilities of zinc-reinforced glass ionomer cement in comparison to traditional high strength glass ionomers. Saudi J. Oral Sci..

[25] Poggio C., Andenna G., Ceci M., Beltrami R., Colombo M., Cucca L. (2016). Fluoride release and uptake abilities of different fissure sealants. J. Clin. Exp. Dent..

[26] Garoushi S., Vallittu P.K., Lassila L. (2018). Characterization of fluoride releasing restorative dental materials. Dent. Mater. J..

[27] Bahsi E., Sagmak S., Dayi B., Cellik O., Akkus Z. (2019). The evaluation of microleakage and fluoride release of different types of glass ionomer cements. Niger. J. Clin. Pract..

[28] Malik S., Ahmed M.A., Choudhry Z., Mughal N., Amin M., Lone M.A. (2018). Fluoride Release From Glass Ionomer Cement Containing Fluoroapatite And Hydroxyapatite. J. Ayub Med. Coll. Abbottabad JAMC.

[29] Hadi M.R. (2020). Effect of increased fluoride contents on fluoride release from glass ionomer cements. Syst. Rev. Pharm..

[30] Panpisut P., Monmaturapoj N., Srion A., Angkananuwat C., Krajangta N., Panthumvanit P. (2020). The effect of powder to liquid ratio on physical properties and fluoride release of glass ionomer cements containing pre-reacted spherical glass fillers. Dent. Mater. J..

[31] Abed F.M., Kotha S.B., AlShukairi H., Almotawah F.N., Alabdulaly R.A., Mallineni S.K. (2022). Effect of Different Concentrations of Silver Nanoparticles on the Quality of the Chemical Bond of Glass Ionomer Cement Dentine in Primary Teeth. Front. Bioeng. Biotechnol..

[32] Lopes C.M.C.D.F., Galvan J., Chibinski A.C.R., Wambier D.S. (2018). Fluoride release and surface roughness of a new glass ionomer cement: Glass carbomer. Rev. De Odontol. Da Unesp.

[33] Madi F., Sidhu S.K., Nicholson J.W. (2020). The effect of temperature and ionic solutes on the fluoride release and recharge of glass-ionomer cements. Dent. Mater..

[34] Lemos J.A., Palmer S.R., Zeng L., Wen Z.T., Kajfasz J.K., Freires I.A., Abranches J., Brady L.J. (2019). The Biology of Streptococcus mutans. Microbiol. Spectr..

[35] Thangavelu L., Veeraragavan G.R., Mallineni S.K., Devaraj E., Parameswari R.P., Syed N.H., Dua K., Chellappan D.K., Balusamy S.R., Bhawal U.K. (2022). Role of Nanoparticles in Environmental Remediation: An Insight into Heavy Metal Pollution from Dentistry. Bioinorg. Chem. Appl..

[36] Yin I.X., Zhao I.S., Mei M.L., Li Q., Yu O.Y., Chu C.H. (2020). Use of Silver Nanomaterials for Caries Prevention: A Concise Review. Int. J. Nanomed..

[37] Degrazia F., Leitune V., Garcia I.M., Arthur R., Samuel S.M.W., Collares F.M. (2016). Effect of silver nanoparticles on the physicochemical and antimicrobial properties of an orthodontic adhesive. J. Appl. Oral Sci..

[38] Naoum S., Ellakwa A., Martin F., Swain M. (2011). Fluoride release, recharge and mechanical property stability of various fluo-ride-containing resin composites. Oper. Dent..

[39] Narang J.K., Narang R.S. (2015). Nanomedicines for dental applications-scope and future perspective. Int. J. Pharm. Investig..

[40] Sreenivasalu P.K.P., Dora C.P., Swami R., Jasthi V.C., Shiroorkar P.N., Nagaraja S., Asdaq S.M.B., Anwer K. (2022). Nanomaterials in Dentistry: Current Applications and Future Scope. Nanomaterials.

[41] Lee D., Kim J., Han M., Shin J. (2020). Fluoride Release and Recharge Properties of Several Fluoride-Containing Restorative Materials. J. Korean Acad. Pedtatric Dent..

[42] Moshaverinia A., Ansari S., Moshaverinia M., Roohpour N., Darr J.A., Rehman I. (2008). Effects of incorporation of hydroxy-apatite and fluoroapatite nanobioceramics into conventional glass ionomer cements (gic). Acta Biomater..

[43] Paiva L., Fidalgo T.K.S., da Costa L.P., Maia L.C., Balan L., Anselme K., Ploux L., Thiré R.M.S. (2018). Antibacterial properties and compressive strength of new one-step preparation silver nanoparticles in glass ionomer cements (NanoAg-GIC). J. Dent..

[44] Itai K., Tsunoda H. (2001). Highly sensitive and rapid method for determination of fluoride ion concentrations in serum and urine using flow injection analysis with a fluoride ion-selective electrode. Clin. Chim. Acta.

[45] Kuşgöz A., Tüzüner T., Ülker M., Kemer B., Saray O. (2010). Conversion degree, microhardness, microleakage and fluoride release of different fissure sealants. J. Mech. Behav. Biomed. Mater..

[46] Rao A., Sudha P. (2011). Fluoride rechargability of a non-resin auto-cured glass ionomer cement from a fluoridated dentifrice: An in vitro study. J. Indian Soc. Pedod. Prev. Dent..

[47] Khurshid Z., Zafar M., Qasim S., Shahab S., Naseem M., AbuReqaiba A. (2015). Advances in Nanotechnology for Restorative Dentistry. Materials.

[48] Amin F., Rahman S., Khurshid Z., Zafar M.S., Sefat F., Kumar N. (2021). Effect of Nanostructures on the Properties of Glass Ionomer Dental Restoratives/Cements: A Comprehensive Narrative Review. Materials.

